# Risk factors and management of atraumatic distal clavicular osteolysis: A scoping review

**DOI:** 10.1177/17585732261479715

**Published:** 2026-08-24

**Authors:** Michael Wilkinson, Nathan Groch, Carrie-Anne Freestone, Archie Clements, Steven Obst

**Affiliations:** 1 School of Health, Medical, and Applied Sciences, 273488 Central Queensland University, Cairns, QLD, Australia; 2 School of Health, Medical, and Applied Sciences, 527836 Central Queensland University, Bundaberg, QLD, Australia

**Keywords:** Osteolysis, clavicle, risk factors, surgical indications

## Abstract

**Introduction:**

The aims of this review were to map the literature for risk factors, clinical and radiographic features of atraumatic distal clavicular osteolysis (ADCO) and indications for surgery.

**Methods:**

A scoping review using PRISMA-ScR guidelines. Four electronic databases [CINAHL, Emcare (Ovid), PubMed, and Scopus] were searched for cohort studies involving ADCO.

**Results:**

Eight studies were included with data on 483 patients. Bench pressing was the most common activity-based risk factor (49.1%) following by general/unspecified weight training (24.4%). Frequency and intensity of loading were noted as potential risk factors for onset of ADCO. The most common reported symptoms were distal clavicle/acromioclavicular joint (ACJ) pain (69.9%). Imaging features included marrow oedema and subchondral fracture of the distal clavicle on MRI. Most patients recovered with conservative care, but 16% required surgery due to failure of conservative care.

**Conclusions:**

Clinical signs and symptoms for ADCO were not distinguishable from other shoulder pathologies. Primary contact clinicians may benefit from consideration of sporting and loading patterns when assessing patients with ACJ pain. MRI was the most frequently utilised imaging modality but should be taken in context with clinical examination. Surgical referral resulted in largely favourable outcomes following resection.

## Background

Distal clavicular osteolysis (DCO) is an often-painful reduction in bone density of the distal end of the clavicle. It typically presents as superior–anterior shoulder pain with focal tenderness over the clavicle and adjacent acromioclavicular joint (ACJ) from either focal trauma or atraumatic repetitive use.^1^ These clinical features overlap substantially with ACJ osteoarthritis (OA), ACJ synovitis, ACJ ligament sprains, and rotator cuff–related shoulder pain, several of which may coexist in overhead or strength sport settings where DCO is prevalent.^2,3^ While the traumatic variant (TDCO) is often more readily identifiable due to a clear inciting event or surgery, atraumatic distal clavicular osteolysis (ADCO) can be more difficult to diagnose as it develops insidiously with signs and symptoms similar to other common atraumatic shoulder pathologies.^2,3^ Without a discrete injury mechanism, targeted imaging may be delayed and a diagnosis of ADCO may be missed, underscoring the need for accurate differentiation from other shoulder pathologies.^4^

Sports considered high-risk for ADCO such as powerlifting and generalized weight training have shown recent upward trends in participation,^5–7^ and engagement in overhead sporting continues to expand, with high and sustained incidence of shoulder injury reported across overhead athlete populations.^8^ Despite an expanding at-risk population, there are no contemporary population-level incidence rates for an increase in ADCO, and historical incidence data are limited. This suggests that ADCO may be going unrecognized in a growing body of athletes, and an hypothesis-driven tool for risk assessment and diagnosis may elucidate the pathology among an undiagnosed cohort.

ADCO is typically diagnosed through a combined clinical and radiological approach. MRI is commonly utilised as an adjunctive imaging modality in ADCO, particularly where clinical suspicion is high, due to its ability to detect distal clavicular bone marrow oedema, subchondral change, and microfracture.^4,9^ In the absence of high clinical suspicion for ADCO, plain radiography is often the first-line imaging modality, despite the condition frequently demonstrating subtle or delayed radiographic changes early in its course. This can further contribute to under-recognition or diagnostic delay by the primary contact clinician.^4,9^ The clinical importance of accurate and timely diagnosis lies in prognosis and treatment selection, as ADCO may fail to respond to standard rehabilitation approaches used for rotator cuff or general ACJ degenerative conditions and, in refractory cases, may require surgical resection of the distal clavicle (DC).^10^ Additionally, a subset of patients will have radiographic and/or symptomatic progression despite conservative management, and may progress to requiring surgical referral in recalcitrant cases.^11^ Earlier differentiation from other shoulder pathologies would then ideally reduce prolonged ineffective rehabilitation and facilitate more targeted imaging and referral pathways.

Although the clinical presentation, imaging features, and risk factors for DCO have been well documented, no study has specifically focused on clinical recognition and early detection of ADCO or when it is appropriate to consider surgical over conservative management. Accordingly, the aims of this review are to map current evidence relating to risk factors for ADCO, determine the most common signs, symptoms, and radiographic features to differentiate it from other shoulder pathologies, and elucidate on when surgical referral is warranted. These multiple aims are directed at helping primary contact clinicians identify patients with ADCO and begin appropriate imaging, treatment and referral pathways.

## Materials and methods

The Preferred Reporting Items for Systematic Reviews and Meta Analysis – Scoping Review (PRISMA-ScR) guidelines were used to structure this scoping review.^12^ Four online databases (CINAHL, Emcare (Ovid), PubMed, and Scopus) were used to source academic articles on the second of May 2025. Each database was searched independently by two authors on the same day, with both returning the same number of articles. The search strategy used a combination of keywords and truncation, including: ‘distal clavicle osteolysis’, ‘distal clavicular osteolysis’, ‘clavicular osteolysis’, ‘osteolysis of the distal clavicle’, and related variations using wildcard operators (e.g., clavic*, osteolysis). Search strategies were adapted for each database, and all searches were conducted on the same day to ensure consistency. No restrictions were placed on search criteria. The inclusion criteria for articles included (1) full-text articles, (2) a patient cohort with ADCO confirmed by medical imaging (MRI or X-ray), and (3) subjects must have had atraumatic/stress induced symptoms. Classification of atraumatic cases was based on reported clinical context, although definitions were not consistently applied across studies.

Exclusion criteria for articles were (a) non-English articles (or unable to be translated to English), (b) articles where the full text was unable to be retrieved, (c) individual case studies and (d) studies describing acute traumatic or post-operative DCO.

Eligible articles were exported from each respective database to Endnote 20.^13^ Article title and abstract were then screened by two authors (NG & CF). If an abstract was not available at that point it was included in the full text review. All full text articles were then screened for inclusion independently by two authors (NG & CF). For articles where a consensus couldn't be reached, a third author (AC) mediated on inclusion/exclusion. Authors of articles without free full text and non-English articles were contacted for full text and/or translated versions. If a response was not provided the article was excluded. All included articles underwent a reference list screening to determine if any further articles met inclusion criteria.

Data from each article were extracted and documented in table form. Items included in data extraction included article title, first author, year of publication, study design, country, setting, baseline patient characteristics, sample size, age, gender, methods (statistical tests used), withdrawals (reason) and intervention pathway and modality (surgical or conservative treatments). In cases where articles included cohorts of both traumatic and ADCO, only data from cases explicitly described as atraumatic or stress-induced were extracted and included in the analysis. Studies in which atraumatic cases could not be clearly distinguished were excluded. Patient data inclusive of clinical signs and symptoms, sport and activity background, and imaging findings were pooled and presented as a percentage of the total number of patients or scans, respectively. The reported percentages should be interpreted as descriptive summaries rather than pooled prevalence estimates and are intended to illustrate general patterns rather than provide precise quantification. Denominators were reported as the total of reported patients or scans available for that outcome.

Methodological quality assessment was conducted using the Joanna Briggs Institute (JBI) Critical Appraisal Tools appropriate to each study design.^14,15^ Given the heterogeneity of study designs, appraisal was used to guide a qualitative assessment of methodological strengths and limitations rather than to generate comparable scores across studies. A checklist was completed independently by two authors (AC & CF) with Inter-Rater reliability calculated using Cohens *κ* = 0.81, indicating substantial agreement.^16^ Differences in interpretation were discussed between reviewers until a consensus was reached. Each JBI checklist was completed using standard response categories (yes/no/unsure/not applicable) to guide qualitative appraisal.

## Results

**Figure 1.**
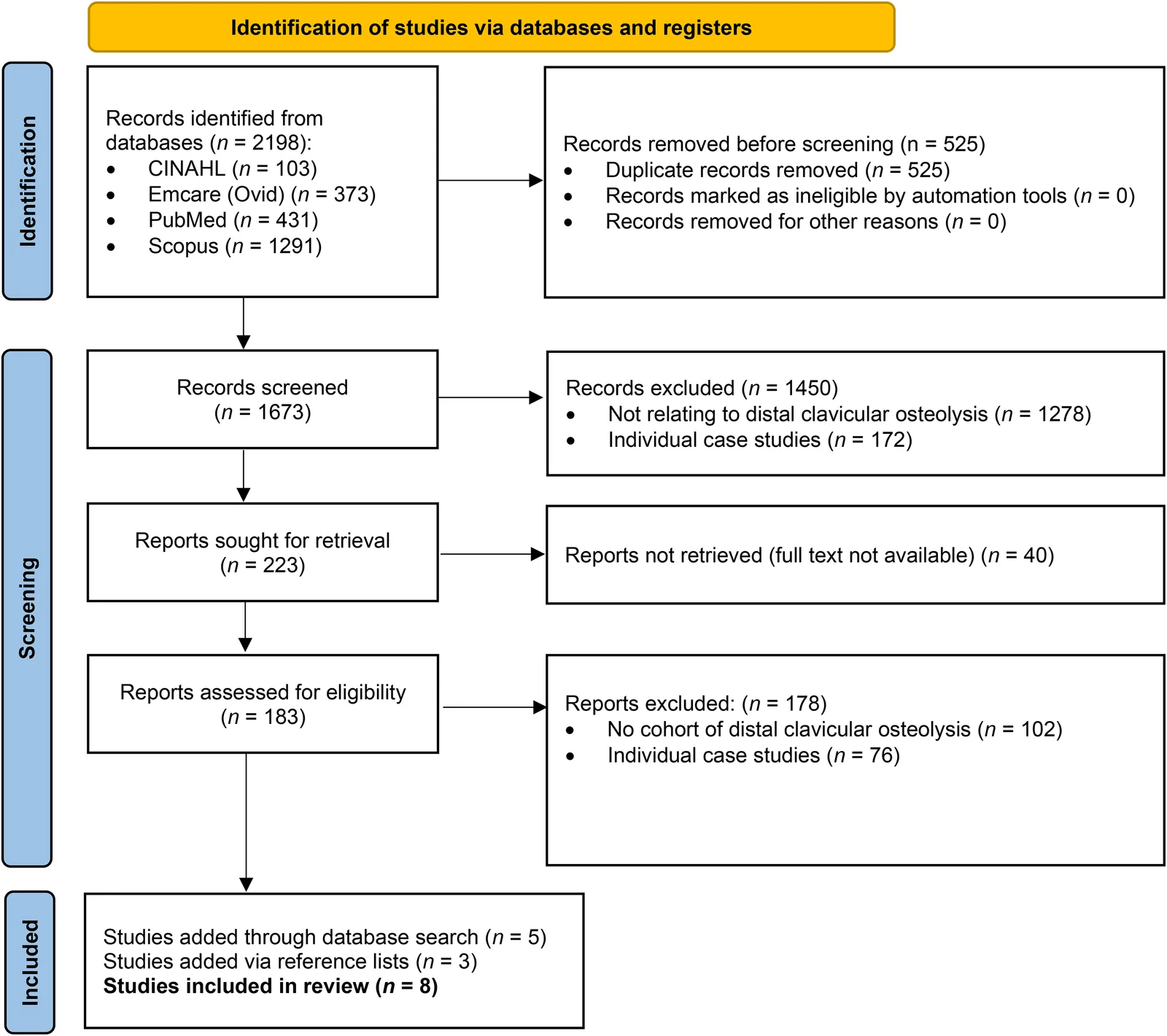
PRISMA-ScR flow diagram.

The initial search identified a total of 2198 records, with 525 duplicates being removed (Figure 1). A total of 1673 articles were assessed for title and abstract screening, of which 1450 were excluded. The remaining 223 articles were then sought for a full-text review. Five studies were included to the review following this process, with the reference lists of these articles being screened and adding a further three articles for a total of eight.

Of the eight studies included there were two case series,^17,18^ two retrospective case series,^19,20^ one retrospective observational cohort study,^21^ two retrospective case control studies^22,23^ and one cross sectional comparative prevalence study.^24^ Across the included studies, 483 patients (454 male and 29 female) and 288 controls were sampled, with a mean age from 15.9 to 39 years (range 13–66 years old). Individual study characteristics are outlined in Table 1. Methodological appraisal identified several consistent limitations across the included studies. Most studies were retrospective in design, with limited reporting of follow-up duration and outcome measures. Sample sizes were often small or heterogeneous, and definitions of clinical improvement and treatment pathways varied considerably. These factors limit the ability to directly compare findings across studies.

**Table 1.** Study characteristics and methodological grading.

Author (year)	Design	Sample size (male/female)
Cahill and Peoria (1982)^1^	Case series	*n =* 46 (46/0)
De la Puente et al. (1999)^20^	Retrospective case series	*n =* 10 (9/1)
Kassarjian et al. (2006)^4^	Retrospective case control	*n =* 36 (32/4)
Mestan and Bassano (2001)^18^	Case series (atraumatic subset)	* n =* 4 (4/0)
Nevalainen (2016)^22^	Retrospective case control	*n =* 262 (262/0)
Patten (1995)^19^	Retrospective case series	*n =* 7 (5/2)
Roedl et al. (2015)^21^	Retrospective cohort	*n =* 93 (71/22)
Scavenius and Iverson (1992)^24^	Cross sectional	*n =* 25 (25/0)

**Figure 2.**
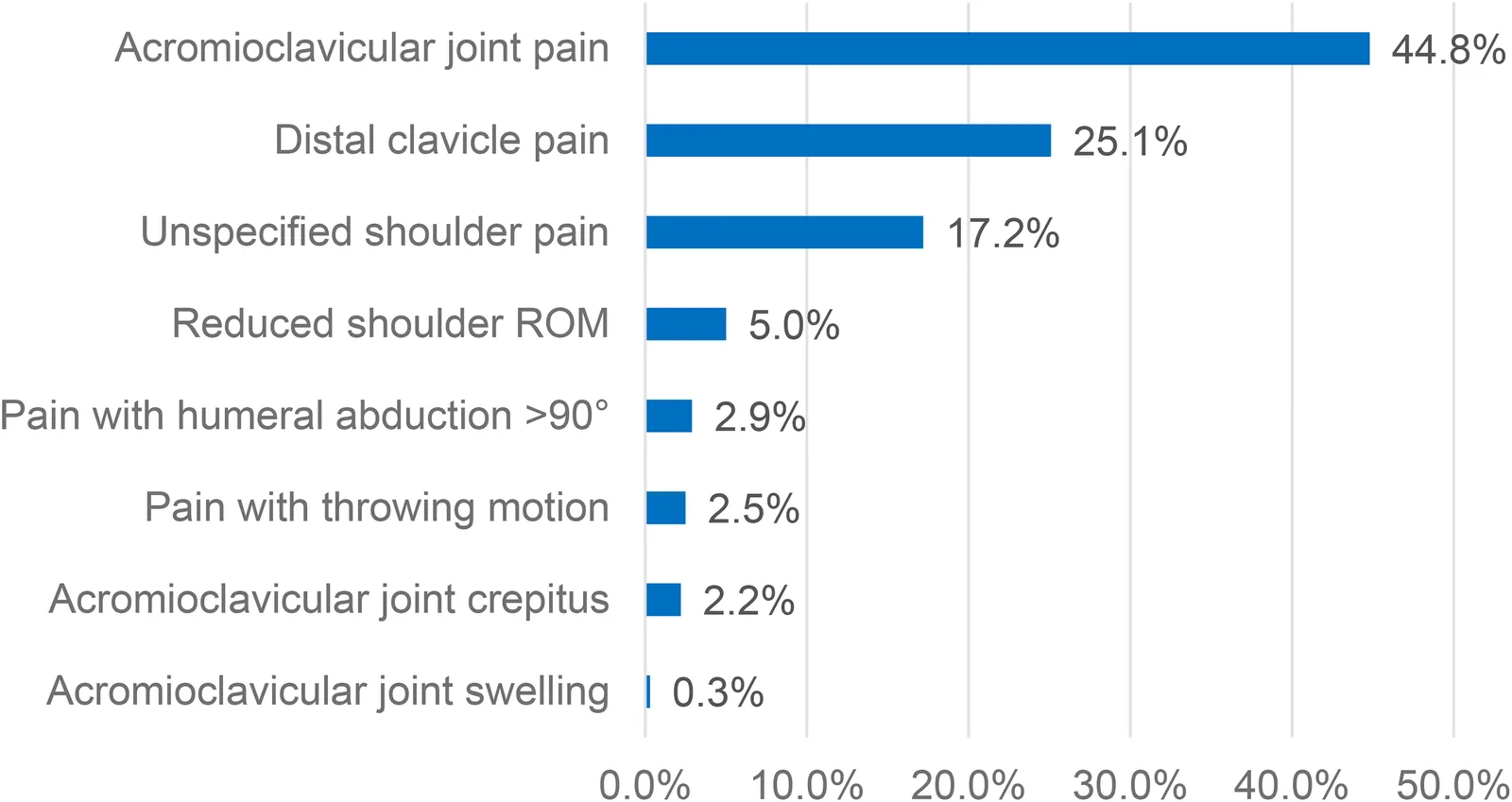
Signs and symptoms of patients with distal clavicular osteolysis (*n =* 279 patients). Categories are not mutually exclusive, and totals may not equal 100% due to multiple reported symptoms/activities per case.

A total of 279 ADCO patients had signs and symptoms available for review. The most prevalent clinical feature was pain, which presented in 87.1% of patients, with 69.9% of patients reporting their pain focal to the ACJ region. Percentages for all signs and symptoms are outlined in Figure 2.

**Figure 3.**
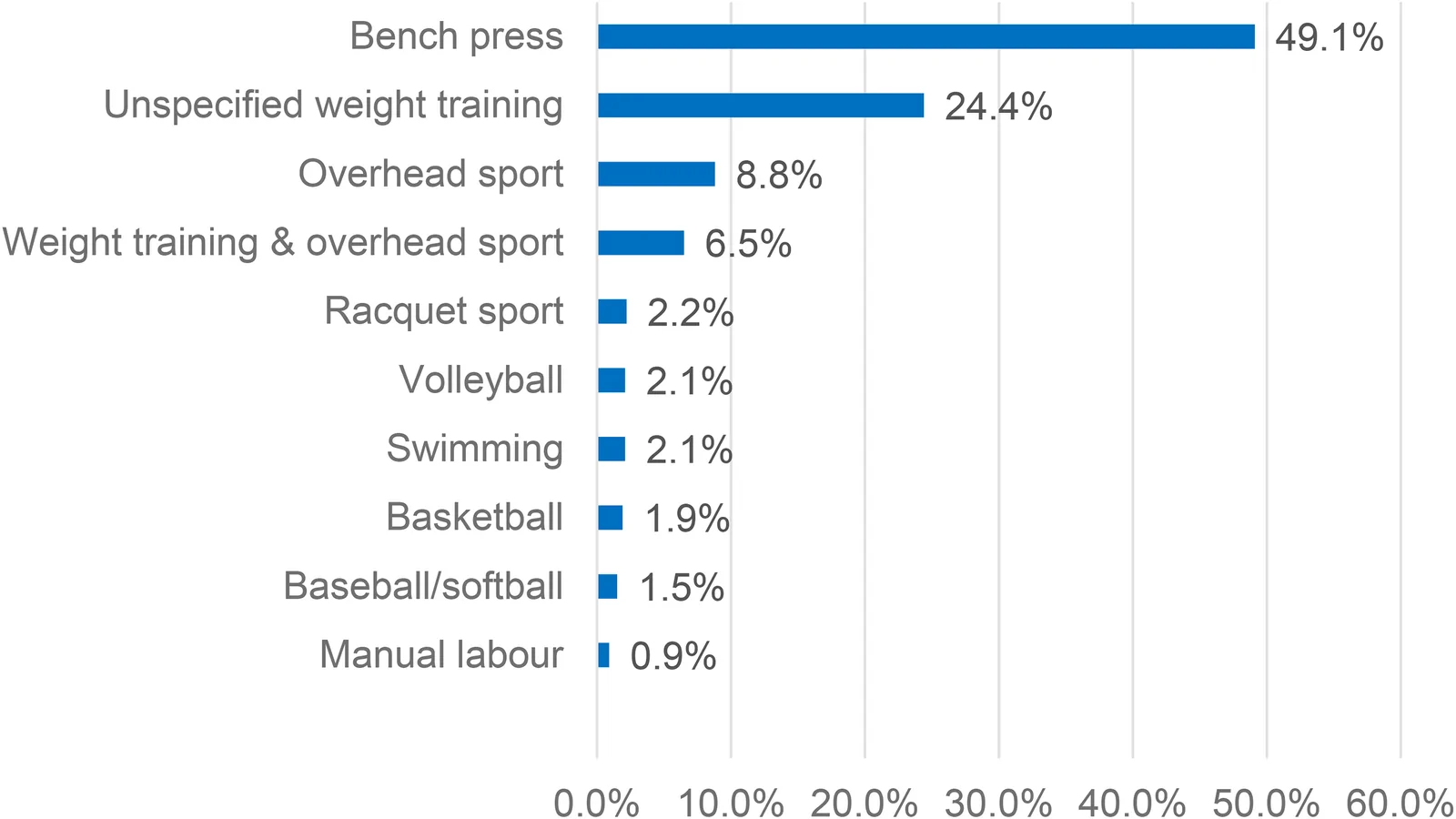
Sport and physical activity background of patients with ADCO (*n =* 483 patients). Categories are not mutually exclusive, and totals may not equal 100% due to multiple reported activities per case.

Recreation, sport, and physical activity background was available for 483 patients and is reported in Figure 3. The most common activity associated with ADCO was bench pressing, accounting for 49.1% of presentations. Bench press training intensity was noted as a risk factor in one study, associating a higher percentage of lifters (56%) acquiring ADCO when pressing greater than 1.5 times bodyweight when compared to controls (6%).^22^ A weight training frequency of three or more times per week was also suggested as having an increased risk for ADCO onset.^17^

**Figure 4.**
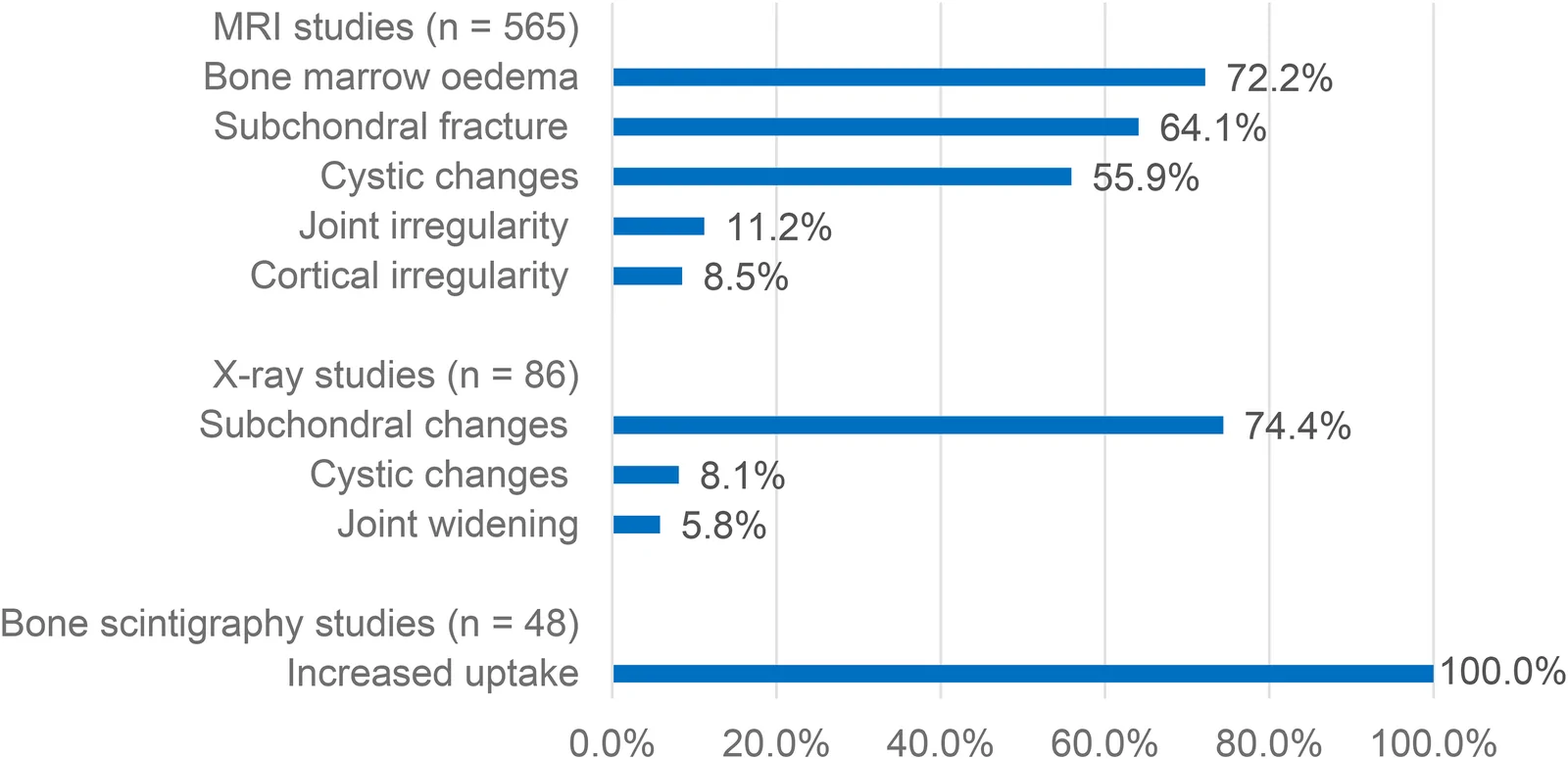
Percentage of imaging findings in ADCO patients. Sample sizes (*n*) refer to number of scans performed among all studies inclusive of repeated scans on the same patient(s).

Across the included studies, MRI was utilized as a diagnostic tool in five studies,^4,19–22^ with two studies using repeat MRI to monitor pathological progression and/or improvement.^19,21^ Plain radiography was used in in four studies,^18–20,24^ and bone scintigraphy in two.^1,19^ MRI identified bone marrow oedema in 72.2% of cases and subchondral fracture in 64.1%, supporting a stress-related microfracture and reparative pathology as the dominant mechanism (Figure 4). Cystic change was also common on MRI (55.9%), whereas overt cortical irregularity (8.5%) and joint irregularity (11.2%) were comparatively infrequent, consistent with MRI detecting earlier intraosseous disease prior to advanced structural breakdown. In contrast, plain radiography primarily demonstrated subchondral changes (74.4%), while secondary features such as cystic change (8.1%) and ACJ widening (5.8%) were uncommon, reflecting the delayed and relatively insensitive nature of radiographs for early stage ADCO. Bone scintigraphy, although infrequently used, showed increased radionuclide uptake in 100% of scanned cases across the cohorts utilizing it, indicating consistently elevated bone turnover at the distal clavicle when this modality was used.^1,19^ It is noted, however, that studies utilizing bone scintigraphy were dated compared to more contemporary research relying on MRI as a more accessible and less invasive imaging option.

Across the included studies, 415 patients were reported to improve with conservative treatment, which included rest^17–20,24^; training modification^17,19^; NSAIDs^17,19,20^; corticosteroid injection^17,20^; sling immobilisation, cryotherapy and ultrasound.^18^ However, definitions of improvement and follow-up durations varied between studies.

Among patients undergoing surgery (*n =* 68), improvement in pain and/or function or return to sport was reported in most cases; however, outcome definitions and duration of follow-up were heterogeneous across studies.^19–22,24^ Conversion to surgery in most reported cases was a result of unsuccessful conservative management, as defined within individual studies. Additional reasons for conversion to surgery included high-grade, structural disease progression (4.4%, *n =* 3) and an unwillingness to modify load due to athletic or occupational constraints (4.4%, *n =* 3). Operative intervention in all cases was open or arthroscopic resection of the distal clavicle, with the most frequent amount of resection documented between 5 and 10 mm.^21,22^ Reported outcomes were generally favourable, although interpretation is limited by inconsistent outcome reporting and the potential influence of surgical factors not captured in the included studies. Of those that converted to surgery, improvement in pain and function, or return to sport was noted in all but five cases.^1^ No subjects required revision or reconstructive surgery following primary resection. It should be noted that treatment pathways and outcomes were not consistently reported across all studies, and these figures reflect aggregated reporting rather than uniformly defined outcome measures.

## Discussion

This scoping review aimed to identify the risk factors for ADCO and outline the most prevalent signs, symptoms and radiographic findings to help differentiate it from other shoulder disorders and determine when to convert to surgery. The screening process identified eight articles which were evaluated using JBI appraisal of quality assessment. Bench pressing, weight training, and overhead athletics were the most frequently reported activity-related risk factors for ADCO across the included studies. More specifically, high-intensity bench pressing greater than 1.5 body weight and upper body weight training more than 3× per week. Occupational exposures were poorly and inconsistently reported across the included studies, representing a limitation of the current evidence base. The most prevalent clinical symptoms of ADCO include ACJ pain, followed by distal clavicle and unspecified shoulder pain. The most common imaging findings include bone marrow oedema, subchondral fracture and erosion of the DC on MRI. It was noted that several included studies predate contemporary imaging and diagnostic standards.^1,19^ Surgery was utilised in 16% of recalcitrant cases, with good outcomes noted among the majority of patients undergoing distal clavicle resection.

This review found that high-intensity bench pressing was the most frequent risk factor for the development of ADCO. Biomechanical analysis of the bench press provides a potential explanatory framework for the association between weight training and ADCO observed in the included studies. During the bench press, the ACJ is exposed to phase-specific mechanical loading that may influence stress-related injury.^25,26^ During the concentric (lifting) phase, compressive forces are transmitted across the ACJ, while during the eccentric (lowering) phase the shoulder is positioned in horizontal abduction and external rotation, generating substantial shear forces and increased tensile loading of the acromioclavicular and coracoclavicular stabilising ligaments.^25,27^ Repetitive exposure to this combined compressive and shear loading profile, particularly under high external loads, may exceed the physiological load tolerance of the distal clavicle and potentially contribute to microstructural failure. Variations in bench press technique, such as grip width, have been shown in biomechanical studies to alter load distribution across the ACJ, and while these findings suggest a potential influence on joint loading, their clinical relevance in the development or prevention of ADCO remains uncertain.^27^

Previous biomechanical modelling has demonstrated that a grip of 1.5 times bi-acromial width reduced ACJ and glenohumeral joint shear forces by approximately 8–21% compared with both narrower (1.0 times) and wider (2.0 times) biacromial grips, without compromising one-repetition maximum performance.^25,28^ Importantly, this grip modification preserved pectoralis major activation while reducing subscapularis and supraspinatus activation, suggesting that alterations in bench press mechanics may reduce detrimental joint loading without substantially altering performance. Collectively, these biomechanical data suggest bench pressing may increase mechanical loading at the ACJ in a manner consistent with proposed stress-related pathology through repetitive high-magnitude shear and compressive loading of the ACJ. These biomechanical findings should be interpreted as hypothesis-generating and provide a theoretical basis for understanding potential mechanisms of ADCO, rather than establishing causation or informing definitive preventative strategies. Prospective studies are required to determine whether these biomechanical factors are independently associated with the development of ADCO or may be modified to influence outcomes.

Clinical signs and symptoms of ADCO captured by this review did not offer useful diagnostic utility for the condition. The predominant clinical feature of ADCO was ACJ or distal clavicle pain, which overlaps with multiple local shoulder pathologies including ACJ OA, synovitis, ligamentous injury, and rotator cuff or labral pathology.^3,29–32^ Given the notable overlap among pathologies with ACJ pain and symptom commonality, the primary contact clinician should consider the key historical and risk factor analysis accompanying the patient with focal ACJ pain. In the presence of historical high-intensity bench pressing or overhead athletics with ACJ pain, ADCO may be considered a differential diagnosis. Once this suspicion is predominant over other differentials, MRI was the most frequently reported advanced imaging modality across the included studies, with common findings including bone marrow oedema, subchondral fracture, and cystic change. Importantly, imaging findings alone do not establish the ACJ as the primary pain generator. Structural abnormalities such as marrow oedema or subchondral change may be present in asymptomatic individuals or coexist with other shoulder pathology. As such, imaging must be interpreted in the context of clinical assessment as structural changes on MRI may not correlate with symptoms and have been reported in asymptomatic individuals.^33^ In clinical practice, the key diagnostic consideration is whether ADCO provides a more plausible explanation for the patient's presentation than other potential pain generators, including ACJ degeneration, synovitis, rotator cuff pathology, or labral injury.

Across the included studies, surgical intervention was generally reported following a trial of conservative management of approximately three months’ duration, although this varied between cohorts.^21,22^ In these instances, surgery was reserved for patients with persistent symptoms, higher-grade MRI findings, and expectations aligned with operative treatment. Accurate localisation of symptoms to the ACJ is an important component of surgical decision-making, although this was not consistently reported across studies. Clinical examination should aim to reproduce patient-specific symptoms with discrete testing of the ACJ and distal clavicle. While specific clinical examination techniques were largely underreported in the included studies and generally do not specifically target ADCO, previous research would suggest the use cross-body abduction^34^ (sensitivity 77%, specificity 79%), O’Brien's active compression test^35^ (sensitivity 6–40%, specificity 90–95%), and discrete ACJ palpation^35^ (sensitivity 96%, specificity 10%) as having diagnostic utility for ACJ pathology. In cases where physical and/or imaging findings are equivocal, or there is a failure of appropriate rehabilitation, anaesthetic injection into the ACJ has been supported as a diagnostic tool to confirm the joint as the primary source of nociception.^36^

While surgical outcomes were generally reported as favourable across the included studies, several important considerations from shoulder surgery practice should be acknowledged. The extent of distal clavicle resection, typically reported between 5–10 mm, must balance adequate decompression with preservation of joint stability, as excessive resection may contribute to horizontal instability.^37^ Preservation of the ACJ capsule is also considered important in maintaining postoperative stability.^38^ Persistent pain following distal clavicle excision has been described and may relate to incomplete resection, capsular disruption, or the presence of unrecognised concomitant pathology.^39^ Coexisting rotator cuff or labral pathology may contribute to ongoing symptoms if not identified and addressed at the time of surgery.^39^ These considerations highlight that surgical outcomes are influenced by a combination of technical, anatomical, and diagnostic factors, many of which were not consistently detailed in the included studies.

**Figure 5.**
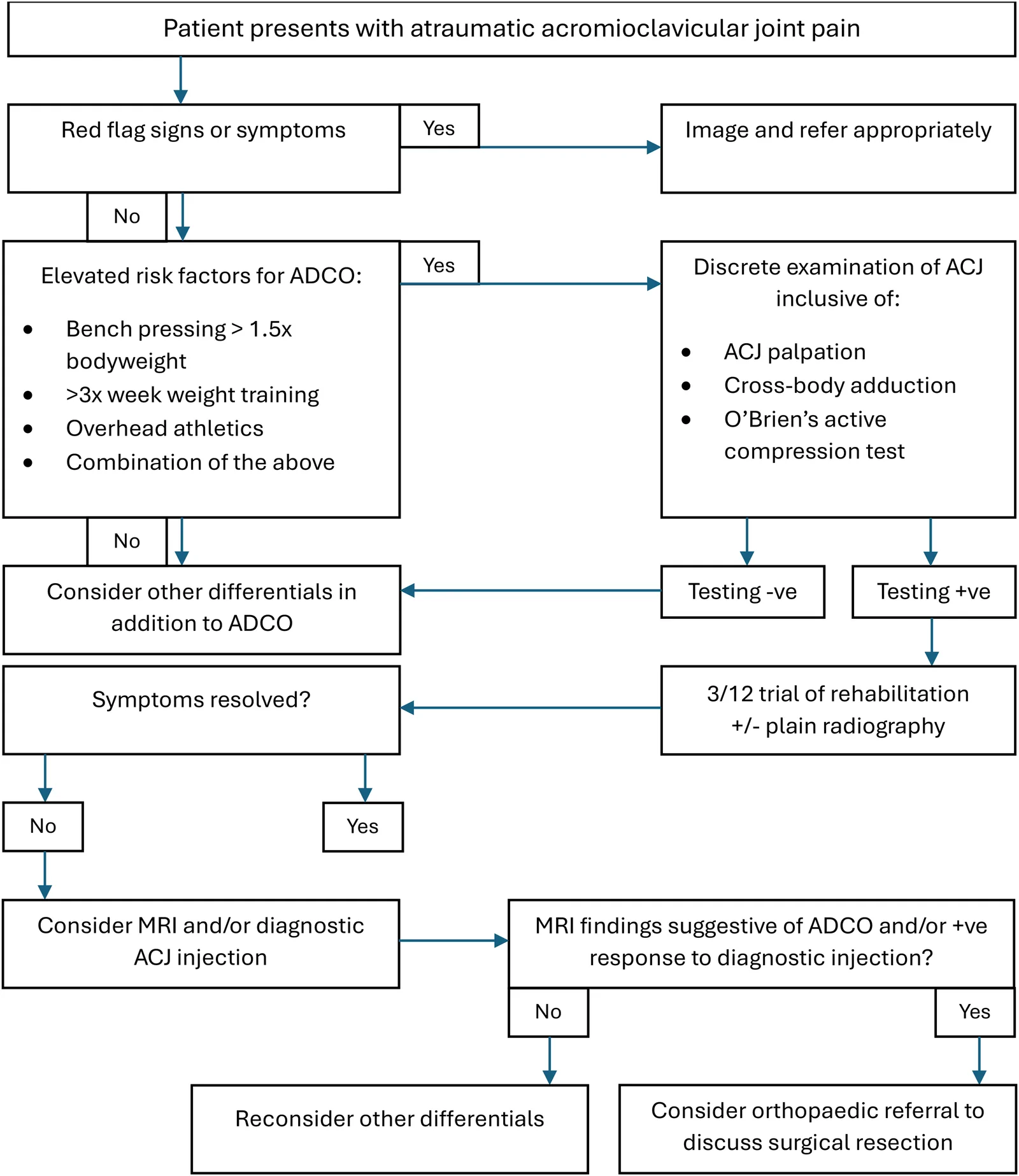
Proposed evidence-informed framework illustrating reported clinical reasoning approaches in ADCO (not validated for clinical decision-making).

Given the overlap in clinical presentation with other shoulder pathologies, diagnosis of ADCO requires careful integration of patient history, physical examination, and imaging findings. Clinical assessment should aim to localise symptoms to the acromioclavicular joint through targeted examination, with imaging used to support the diagnosis when suspicion remains high. Diagnosis also requires determination of whether examination and imaging results best explain the patient's symptoms when compared to other potential sources of shoulder pain. Where multiple potential pain generators are identified, prioritisation should be guided by symptom reproduction, load-related behaviour, and response to targeted clinical tests or diagnostic interventions. Based on the findings of this review and existing data on ADCO and ACJ pathology, we propose a hypothesis-generating, evidence-informed framework (Figure 5) to illustrate how the reported factors may be integrated into clinical reasoning. This framework is intended to guide future research and should not be interpreted as a validated clinical pathway.

The strength of this systematic scoping review is its methodologic rigor. The primary limitations relate the nature of the included studies, as they were largely retrospective with several being dated compared to more contemporary cohorts on ADCO. Methodological appraisal identified consistent limitations across studies, including retrospective designs, small or heterogeneous samples, and inconsistent outcome reporting, which limits the strength and generalisability of the findings. This in turn limits the strength of the conclusions from this review. While our flowchart outlines a proposed framework for the assessment and treatment of ADCO, prospective validation would be needed to improve the overall model of care proposed. Moving forward, specific cohort data on strength and overhead athletics may capture a higher prevalence of ADCO given the growth in participants. Importantly, the scoping design and the heterogeneity of included studies limit the ability to make definitive clinical recommendations, and the findings should be interpreted as descriptive rather than prescriptive.

## Conclusion

ADCO is an uncommon condition that presents with focal ACJ pain and demonstrates substantial clinical overlap with other shoulder pathologies. Across the included literature, participation in weight training and overhead sport was frequently reported, and MRI was commonly used to identify features such as bone marrow oedema and subchondral fracture. The findings of this review highlight the importance of patient history and loading patterns in raising clinical suspicion of ADCO. Conservative management was commonly trialled as a first-line approach, with a subset of patients progressing to surgical intervention following persistent symptoms. Given the retrospective and heterogeneous nature of the available evidence, these findings should be interpreted as descriptive. Further prospective research is required to establish optimal diagnostic and management pathways.
